# Disparity in clinical characteristics between 2019 novel coronavirus pneumonia and leptospirosis

**DOI:** 10.1515/med-2021-0262

**Published:** 2021-03-30

**Authors:** BinBin Li, ChunMiao Bao

**Affiliations:** Department of Respiratory Medicine, YongJia County People’s Hospital, Wenzhou, China; Department of Respiratory Medicine, The Affiliated Yangming Hospital of Ningbo University, Yuyao People’s Hospital of Zhejiang Province, Yuyao, China

**Keywords:** COVID-19, leptospirosis, epidemiology, computed tomography, typhoon

## Abstract

**Objective:**

A cluster outbreak of patients with similar symptoms and computed tomographic (CT) images of COVID-19 were diagnosed with leptospirosis. This study was aimed to identify the clinical difference between leptospirosis and COVID-19, providing evidence for strategy optimization.

**Methods:**

A cohort of leptospirosis patients were collected and compared with age- and gender-matched COVID-19 cases in the epidemiological investigation, chest CT scan, laboratory tests, and length of hospital stay.

**Results:**

Compared with COVID-19, contacting floodwater and lack of family clustering were features of leptospirosis in epidemiological assessment. In the laboratory test, higher level of white blood cells (WBCs: (10.38 ± 4.56) × 109/L vs (6.45 ± 1.95) × 109/L, *p* < 0.001), C-reactive protein (CRP: (138.93 ± 73.03) mg/L vs (40.28 ± 30.38) mg/L, *p* < 0.001), Creatine ((88.27 ± 35.16) mmol/L vs (63.31 ± 14.50) mmol/L, *p* < 0.001), and a lower level of platelet ((152.93 ± 51.93) × 109/L vs (229.65 ± 66.59) × 109/L, *p* < 0.001) were detected on patients with leptospirosis.

**Conclusion:**

Given the epidemiological differences and seasonal prevalence, it is important to suspect leptospirosis in cases with a similar presentation of COVID-19. The clinical disparities may facilitate the therapeutic management of these two diseases.

As a major threat to public health, the climbing number of COVID-19 cases is increasing globally [1]. Cluster infections are a common feature of this disease [2]. Recently, there was a clustered outbreak of patients with similar manifestations (fever and computed tomographic (CT) image of lung lesion) as COVID-19, and they were admitted to our medical centers and subsequently diagnosed with leptospirosis. Due to the infection control precautions for COVID-19, tests and treatments for febrile diseases (such as leptospirosis) are often carried out after the COVID tests return negative. Given the 12–24 h of turn-around time for COVID-19 tests, the diagnosis and the treatment for febrile diseases may often be delayed. Considering the overlap in presentation, shunting of medical resources, and often mandatory precautions in suspected COVID-19 may cause a significant delay in diagnosis and the treatment of leptospirosis. The aim of this study is to identify the potential disparities between leptospirosis and COVID-19, providing evidence for strategy optimization.

## 1 Methods

Cases of leptospirosis (*n* = 41) were recruited from two tertiary hospitals in Zhejiang Province, from July 1, 2020, to September 1, 2020. By using real-time PCR (qPCR), these cases were confirmed with a diagnosis of leptospirosis by the center for disease control and prevention (CDC). Most leptospirosis patients had directly contacted flood or worked in farmland barefoot after the typhoon. Demographic data, laboratory tests (including levels of white blood cell, neutrophils, eosinophils, hemoglobin, platelet, C-reactive protein (CRP), creatine, and creatine kinase), and chest CT scan during the prehospitalization period were collected and compared with a cohort of 57 moderate COVID-19 cases. Also, the length of hospital stay (LOS) was compared between these two groups. The approval was obtained from the ethics committee, and the consent was waived.

Normally distributed continuous data were expressed in terms of mean ± standard deviation (SD), whereas continuous data with nonnormal distribution were expressed in terms of the median (interquartile range). Categorical data were expressed in terms of frequency (percentage). Differences between groups were evaluated using independent sample *t*-test, Wilcoxon signed-rank tests, and chi-square test. *P* < 0.05 was defined as statistically significant.

## 2 Results

Epidemiological investigation showed that the first wave of COVID-19 was mainly transmitted during spring (January and February) [3], whereas leptospirosis cases were admitted for hospitalization during typhoon season (August and September). The differences in epidemiological trace between COVID-19 and leptospirosis were presented in Table 1. Visiting epi-center was commonly found among imported COVID-19 cases. Locally transmitted cases shared a close contact with the case-patient. On the contrary, the path tracking revealed no crossing path among leptospirosis cases. However, the behavior tracking reported that most patients with leptospirosis had contacted floodwater, shortly after a typhoon rising. Another noteworthy difference was that COVID-19 cases merged on a family clustering base (kinship was identified on 26 of 57 patients with COVID-19), whereas no kinship was identified among leptospirosis cases.

**Table 1:** Epidemiological investigation

	COVID-19	Leptospirosis
Time of outbreak	Spring (January and February)	Typhoon season (August and September)
Path tracking	Epi-center	No crossing path
	Direct contact with case-patient	
Family clustering	Common	Rare

All patients in this study experienced fever. In addition, cough (*n* = 58) and sore throat (*n* = 34) were other two common symptoms (Table 2). Compared with COVID-19, more leptospirosis patients experienced the symptom of muscle pain (19 [33.33%] vs 26 [63.41%], *p* = 0.003; Table 2). For the initial CT screen, a lesion was found on each case. While assessing by the location of the lesion (unilateral lung lesion vs bilateral lung lesions), more than half of leptospirosis cases (*n* = 27, 65.85%) and COVID-19 cases (*n* = 31, 54.39%) had bilateral lung lesions (Table 2). However, the difference between the two groups was not statistically significant (*p* = 0.225). Compared with leptospirosis patients, a marginally significant longer LOS was found in COVID-19 patients ((12.35 ± 4.94) days vs (10.26 ± 4.10) days, *p* = 0.059; Table 2).

**Table 2:** Disparity in clinical characteristics between COVID-19 and leptospirosis

	Total	COVID-19	Leptospirosis	*p* value
	(*N* = 98)	(*N* = 57)	(*N* = 41)	
**Demography**				
Age^a^ (years)	56.00 (12.70)	53.26 (10.95)	58.07 (13.65)	0.112
Male^b^	60 (61.22)	32 (56.14)	28 (68.29)	0.456
**Symptom** b				
Cough	58 (59.18)	35 (61.40)	23 (56.10)	0.598
Sore throat	34 (34.69)	23 (40.35)	11 (26.83)	0.165
Muscle pain	45 (45.92)	19 (33.33)	26 (63.41)	0.003
**Laboratory test**				
White blood cell^a^ (10^9^/L)	8.69 (4.14)	6.45 (1.95)	10.38 (4.56)	<0.001
Neutrophils^a^ (10^9^/L)	6.43 (4.96)	1.38 (0.46)	9.14 (4.05)	<0.001
Eosinophils^c^ (10^9^/L)	0.11 (0.21)	0.11 (0.15)	0.11 (0.24)	0.108
Hemoglobin^a^ (g/L)	129.92 (14.54)	131.67 (17.02)	129.02 (13.24)	0.503
Platelet^a^ (10^9^/L)	185.96 (69.69)	229.65 (66.59)	152.93 (51.93)	<0.001
C-reactive protein^a^ (mg/L)	96.45 (76.25)	40.28 (30.38)	138.93 (73.03)	<0.001
Creatine^a^ (mmol/L)	77.52 (30.66)	63.31 (14.50)	88.27 (35.16)	<0.001
Creatine kinase^c^ (µmol/L)	58.50 (68.00)	45.00 (40.00)	65.00 (194.00)	0.049
**Chest CT scan** b				0.225
Unilateral	40 (40.82)	26 (45.61)	14 (34.15)	
Bilateral	58 (59.18)	31 (54.39)	27 (65.85)	
**Length of stay** a (days)	11.20 (4.59)	12.35 (4.94)	10.26 (4.10)	0.059

Abbreviations: SD, standard deviation; IQR, interquartile range; CT, computed tomographic.

a Mean (SD).

b *n* (%).

c Median (IQR).

Laboratory tests also showed differences between COVID-19 and leptospirosis cases (Table 2). Compared with COVID-19 cases with comparable demographic characteristics, the leptospirosis patients had higher level of white blood cells (WBCs: (10.38 ± 4.56) × 10^9^/L vs (6.45 ± 1.95) × 10^9^/L, *p* < 0.001), neutrophils (NEU: (9.14 ± 4.05) × 10^9^/L vs (1.38 ± 0.46) × 10^9^/L, *p* < 0.001), CRP (CRP: (138.93 ± 73.03) mg/L vs (40.28 ± 30.38) mg/L, *p* < 0.001), creatine ((88.27 ± 35.16) mmol/L vs (63.31 ± 14.50) mmol/L, *p* < 0.001), and creatine kinase (CK: 65.00 (194.00) µmol/L vs 45.00 (40.00) µmol/L, *p* = 0.049) but a lower level of platelets ((152.93 ± 51.93) × 10^9^/L vs (229.65 ± 66.59) × 10^9^/L, *p* < 0.001).

A combination of interferon, lopinavir, and Arbidol was used to treat COVID-19 cases, whereas the antibiotics were prescribed to leptospirosis patients, resulting in full recovery on each patient. During the hospitalization, hemoptysis was found on three leptospirosis patients and acute renal dysfunction was identified on another nine leptospirosis cases, two of which had urgent hemodialysis.

## 3 Discussion

While COVID-19 kept spreading [4], a small outbreak of leptospirosis with similar clinical presentations of this pandemic was identified. Given the increasingly likely of false-positive COVID-19 test results in the current epidemiological climate [5], misdiagnosis of COVID-19 may lead to substantial consequences of unnecessary exposure, delayed treatment, and clinical deterioration.

For such a group of patients who were suspected to have COVID-19, the epidemiological report may provide a diagnostic clue for leptospirosis. The previous study showed that the cases associated with water consumption and environmental disasters (typhoons in our case) may likely be leptospirosis outbreaks [6]. Also, family clustering cases were more likely to be COVID-19, suggesting a possible person-to-person transmission. For a potential COVID-19 case, it is critical to identify “who” has this person contacted with, whereas “what” has this person contacted would be a more reasonable question for an individual who was suspected to have leptospirosis.

Being the most accurate testing, qPCR serves as the bona fide standard for COVID-19 in the pandemic. However, healthcare workers in resource-limited situations often diagnose patients presumptively based on clinical features. In our study, laboratory tests may help differentiate COVID-19 from leptospirosis, facilitating the preliminary screening and immediate processing. We found a higher level of WBC, NEU, and CRP in patients with leptospirosis, while the average level of these biomarkers on COVID-19 cases remained in the relatively normal range. However, the increased inflammatory biomarkers were reported in severe COVID-19 cases [7], suggesting that the founding of this study may not be applied to COVID-19 patients with more severe conditions.

From the therapeutic perspective, the treatment for COVID-19 may have potential clinical harm to leptospirosis, pending the current stage of the disease. COVID-19 therapies of biologicals may suppress the cytokine response, which is essential for the early elimination of leptospirosis pathogens [8]. However, for uncontrolled cytokine storm in both COVID-19 and severe leptospirosis, biologicals may be helpful [9]. Based on laboratory tests and CT scans, rare severe cases of leptospirosis developed in our study, suggesting that cautiousness should be taken on the therapeutic strategy. In addition, increased levels of CK and creatine were found in the leptospirosis cases. CK is commonly used as a measure of muscle damage, which triggers muscle pain, consisting of the difference we found in symptoms. Compared to moderate COVID-19, an increased level of creatine was found in leptospirosis cases, suggesting various degrees of renal damage, which ultimately warranted urgent hemodialysis for two leptospirosis patients. In situations with shortages of staff and supplies, the ability to provide kidney replacement therapy may be pivotal for leptospirosis cases.

Our study highlights the importance that the healthcare workers should be sensitized about the importance of suspecting leptospirosis in COVID-19 suspects. There is a need for formulating integrated clinical strategies for processing cases with a similar presentation of COVID-19, keeping into account the epidemiology and seasonal prevalence of leptospirosis. Confounders such as smoking and drinking left unadjusted, and the relatively small sample size may be the main limitations of this study. Nevertheless, this study provided some key points for the diagnosis and the treatment for leptospirosis as a COVID-19 mimic, which may contribute to the distribution optimization in the medical resources.
